# A Novel Role for Transcription Factor *Lmo4* in Thymus Development Through Genetic Interaction with *Cited2*

**DOI:** 10.1002/dvdy.22334

**Published:** 2010-05-28

**Authors:** Anna C Michell, José Bragança, Carol Broadbent, Bradley Joyce, Angela Franklyn, Jürgen E Schneider, Shoumo Bhattacharya, Simon D Bamforth

**Affiliations:** Department of Cardiovascular Medicine, Wellcome Trust Centre for Human Genetics, University of OxfordRoosevelt Drive, Oxford, United Kingdom

**Keywords:** *Cited2*, *Lmo4*, thymus development, *Tbx1*, modifiers

## Abstract

Deletion of the transcriptional modulator *Cited2* in the mouse results in embryonic lethality, cardiovascular malformations, adrenal agenesis, cranial ganglia fusion, exencephaly, and left–right patterning defects, all seen with a varying degree of penetrance. The phenotypic heterogeneity, observed on different genetic backgrounds, indicates the existence of both genetic and environmental modifiers. Mice lacking the LIM domain-containing protein *Lmo4* share specific phenotypes with *Cited2* null embryos, such as embryonic lethality, cranial ganglia fusion, and exencephaly. These shared phenotypes suggested that *Lmo4* may be a potential genetic modifier of the *Cited2* phenotype. Examination of *Lmo4*-deficient embryos revealed partially penetrant cardiovascular malformations and hypoplastic thymus. Examination of *Lmo4;Cited2* compound mutants indicated that there is a genetic interaction between *Cited2* and *Lmo4* in control of thymus development. Our data suggest that this may occur, in part, through control of expression of a common target gene, *Tbx1*, which is necessary for normal thymus development. Developmental Dynamics 239:1988–1994, 2010. © 2010 Wiley-Liss, Inc.

## INTRODUCTION

The transcription factor CITED2, which binds CREBBP with high affinity (Bhattacharya et al., 1999), acts as a co-factor for transcription factors such as TFAP2, LHX2, PPARA, and SMAD2/3 (Glenn and Maurer, 1999; Braganca et al., 2003; Tien et al., 2004; Chou and Yang, 2006), as well as acting as a repressor of hypoxia-activated transcription (Bhattacharya et al., 1999). Mice null for *Cited2* die in utero from phenotypically heterogeneous cardiac malformations including atrial, ventricular, and atrioventricular septal defects (ASD, VSD, AVSD); outflow tract defects (double-outlet right ventricle [DORV], common arterial trunk [CAT], tetralogy of Fallot [TOF], transposition of great arteries [TGA]); and interrupted and right-sided aortic arch (Bamforth et al., 2001, 2004; Yin et al., 2002; Weninger et al., 2005). Loss of *Cited2* also gives rise to exencephaly, cranial ganglia fusion, abnormal migration of cardiac neural crest cells, absent adrenal glands, and placental abnormalities (Bamforth et al., 2001, 2004; Barbera et al., 2002; Yin et al., 2002; Weninger et al., 2005; Withington et al., 2006; Val et al., 2007). On a congenic C57Bl/6J background, *Cited2*-null mice show left–right patterning defects characterized by right atrial and pulmonary isomerism, and abnormal ventricular topology (Bamforth et al., 2004; Weninger et al., 2005). We have recently shown that the phenotypically heterogenous and penetrant cardiac malformations in *Cited2* deficiency arise from a primary requirement in epiblast derivatives for left–right patterning, with a secondary cell-autonomous role in the mesoderm (MacDonald et al., 2008).

Cited2 binds the LIM homeodomain transcription factor, Lhx2, by means of LIM domains (Glenn and Maurer, 1999). Proteins containing LIM domains function as transcriptional regulators, and this domain may act as a molecular adaptor for multiprotein complexes. The subfamily of LIM-only (LMO) proteins comprises four members (Lmo1–Lmo4). These non-DNA binding transcription factors may affect transcription by protein–protein interaction with DNA-binding transcription factors or chromatin modeling proteins (Hahm et al., 2004). *Lmo4* comprises two zinc-binding LIM domains and is widely expressed in the developing embryo, including the pharyngeal arches, thymus, and neural crest (Grutz et al., 1998; Kenny et al., 1998; Sugihara et al., 1998). Mouse embryos lacking *Lmo4* display a phenotype that overlaps with *Cited2*^−/−^ embryos. This includes exencephaly, in utero or perinatal lethality, and cranial nerve fusions (Hahm et al., 2004; Tse et al., 2004; Lee et al., 2005).

The shared specific phenotypes observed in *Cited2* and *Lmo4* mutant mice suggest that there may be a genetic interaction. In this study, we show that embryos lacking *Lmo4* have partially penetrant cardiac malformations including VSD, DORV, and right-sided aortic arch, which have not previously been reported. The shared specific phenotypes are further extended by demonstrating cervical fusions and abnormal thymus in *Cited2* and in *Lmo4* mutant embryos. The thymus is significantly smaller in compound mutant embryos, and completely absent in embryos lacking both *Lmo4* and *Cited2*, indicating that there is a genetic interaction. Expression of *Tbx1*, which is important for thymus development, was significantly reduced in *Lmo4* and *Cited2* mutant embryos. This suggests a novel role for *Cited2* and *Lmo4* acting, likely in part through *Tbx1*, to control development of the thymus.

## RESULTS

### Cervical Vertebrae Abnormalities in *Cited2*^−/−^ Embryos

**Figure 1:**
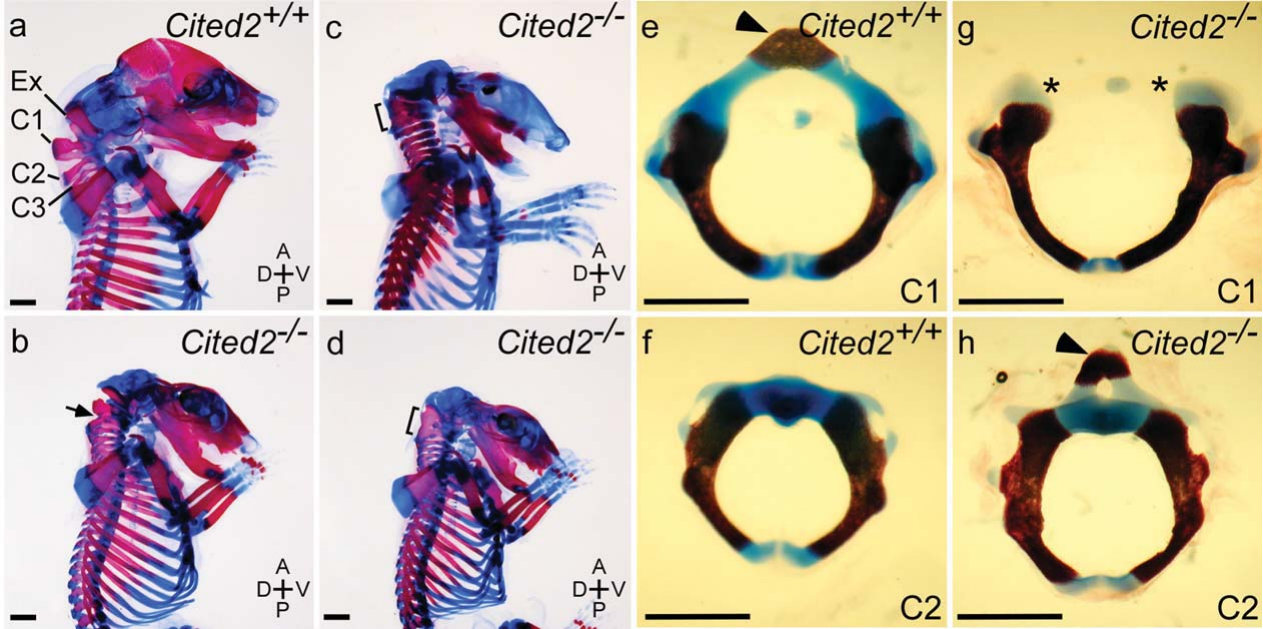
Skeletal defects in embryonic day (E) 17.5 *Cited2*^−/−^ embryos. **a:** Wild-type (*Cited2*^+/+^) embryo skeleton showing the normal appearance of the cervical vertebrae. **b–d:***Cited2*^−/−^ skeletons showing various degrees of fusion between the cervical vertebrae (indicated). All embryos depicted had exencephaly and are missing the occipital and frontal skull bones. e–h: Disarticulated cervical vertebrae from a *Cited*^−/−^ skeleton with less severe fusions. **e:** Wild-type atlas, with anterior arch indicated (arrowhead). **f:** Wild-type axis. **g:***Cited*^−/−^ atlas which is missing the anterior arch (asterisks). **h:***Cited2*^−/−^ axis, which has a structure resembling the anterior arch of the atlas (arrowhead). Ex, exoccipital; C1, atlas; C2, axis. Scale bar = 1 mm.

*Lmo4*^−/−^ embryos show homeotic-like transformations in the rib cage and cervical vertebrae, although at low penetrance (Hahm et al., 2004). To investigate whether similar defects were present in the *Cited2*^−/−^ embryos, skeletal preparations were made at embryonic day (E) 17.5. This revealed that *Cited2*^−/−^ embryos (on a mixed genetic background) have fusions of the cervical vertebrae (Fig. 1). The cervical vertebrae may be completely fused (Fig. 1c,d) from the exoccipital bone through to the atlas (C1), axis (C2), and the third cervical vertebra (C3). Less severe fusions are also observed, which may even be asymmetric (Fig. 1c). The cervical skeleton from one of the less severely affected *Cited2*^−/−^ embryos was disarticulated revealing an anterior transformation (Fig. 1g,h). The axis had a structure resembling the anterior arch of the atlas, and the atlas was missing this. The rib cages, however, were unaffected. Similar cervical vertebrae fusions were also observed in embryos on congenic 129Sv and C57Bl/6J genetic backgrounds (data not shown).

### Cardiovascular Defects and Thymus Hypoplasia in *Lmo4*^−/−^ Embryos

**Figure 2:**
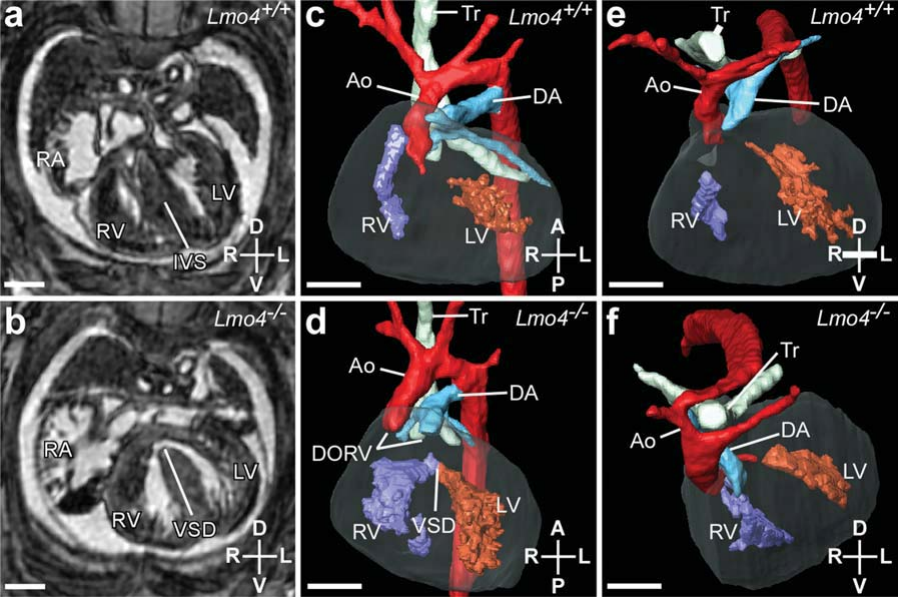
Cardiovascular defects observed in embryonic day (E) 15.5 *Lmo4*^−/−^ embryos. **a,b:** Transverse magnetic resonance imaging sections of wild-type (a) and *Lmo4*^−/−^ (b) embryo hearts. A ventricular septal defect (VSD) is visible in the *Lmo4*^−/−^ heart. c–f: Three-dimensional reconstructions of embryo hearts and great vessels. **c,d:** Ventral views of wild-type embryo heart (c) showing normal topology, and *Lmo4*^−/−^embryo heart (d) showing a VSD and double-outlet right ventricle (DORV), where both the aorta (Ao) and ductus arteriosus (DA) arise from the right ventricle (RV). **e,f:** Anterior views of wild-type embryo heart (e) showing normal topology, and *Lmo4*^−/−^embryo heart (f) showing a right-sided aortic arch and ductus arteriosus. RA, right atrium; LA, left atrium; LV, left ventricle; IVS, intra-ventricular septum; PA, pulmonary arteries; Tr, trachea. Scale bar = 500 μm.

Cardiovascular defects, a major aspect of the *Cited2* phenotype, have not been reported in *Lmo4* mutant mice. We therefore investigated whether the *Lmo4*^−/−^ embryos suffered from any cardiac developmental abnormalities. Heterozygous *Lmo4* mice (Tse et al., 2004) were intercrossed to analyze embryos at E15.5 by magnetic resonance imaging (MRI). Gross external analysis of *Lmo4*^−/−^ embryos showed exencephaly (2/8; data not shown), as previously reported (Hahm et al., 2004; Tse et al., 2004; Lee et al., 2005), and edema (2/8; data not shown). Analysis by MRI revealed incomplete penetrance of cardiovascular defects in *Lmo4*^−/−^ embryos, including VSD with DORV (2/8) and a right-sided aortic arch (1/8; Fig. 2).

**Figure 3:**
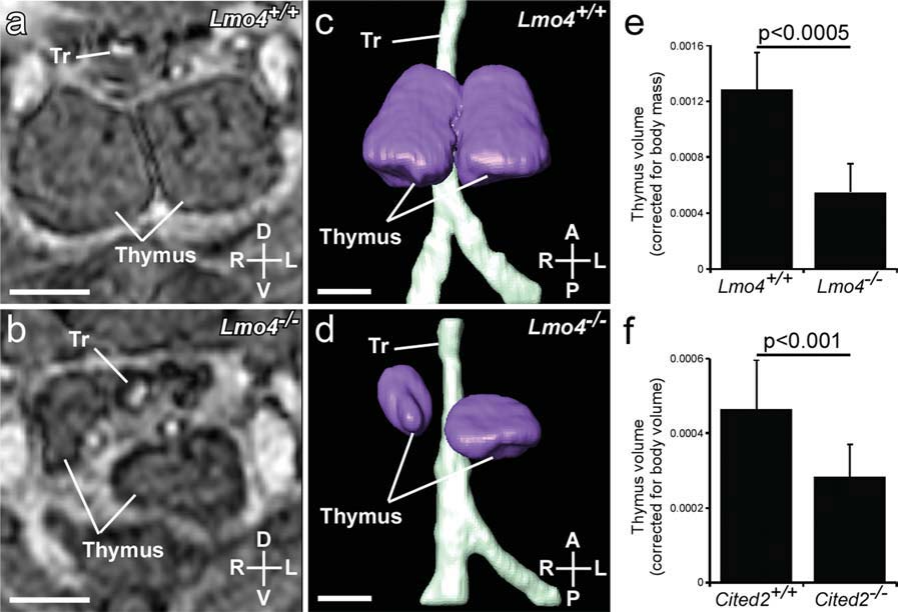
Thymus hypoplasia and malpositioning observed in embryonic day (E) 15.5 *Lmo4*^−/−^ embryos. **a,b:** Transverse magnetic resonance imaging (MRI) sections of the thymus of wild-type (*Lmo4*^+/+^; a) and *Lmo4*^−/−^ (b) littermate embryos. **c,d:** Three-dimensional (3D) reconstructions (ventral view) of thymus and trachea showing normal position and equal-sized lobes from wild-type (c) and hypoplasia, abnormal positioning and separation of the lobes in a *Lmo4*^−/−^ embryo (d). **e:** Thymus volumes from *Lmo4*^+/+^ and *Lmo4*^−/−^ embryos, corrected for embryo mass. **f:** Thymus volumes from *Cited2*^+/+^ and *Cited2*^−/−^ embryos, corrected for whole embryo volume. (Graph values are means ± SD). Scale bar = 500 μm.

*Lmo4*^−/−^ embryos also had consistently hypoplastic thymuses (8/8) compared with wild-type littermates which, in some cases, were malpositioned (3/8, Fig. 3b,d). Volume calculations showed a significant 2.3-fold decrease between wild-type (n = 6) and *Lmo4*^−/−^, thymuses (n = 8; *P* < 0.0005; Fig. 3e). Thymuses of *Lmo4*^+/−^ littermates were not significantly different to those of wild-type littermates (n = 7; data not shown). Thymus size in *Cited2*^−/−^ embryos at E15.5 was then assessed to see if they were affected. A significant 1.6-fold decrease in thymus volume was found (n = 6 wild-type, n = 6 *Cited2*^−/−^; *P* < 0.001; Fig. 3f). We also measured adrenal gland volume, because *Cited2*^−/−^ embryos exhibit adrenal agenesis (Bamforth et al., 2001). However, we did not find any significant reduction in the size of adrenal glands in *Lmo4*^−/−^ embryos compared with those of wild-type littermates (n = 6 for each genotype; data not shown).

### *Cited2* and *Lmo4* Genetically Interact In Vivo

As *Cited2* and *Lmo4* mutant mice share several specific phenotypes, we tested whether there was a genetic interaction in vivo by intercrossing *Lmo4*^+/−^ mice with *Cited2*^+/−^ mice (Supp. Table S1, which is available online). As *Lmo4*^+/−^;*Cited2*^+/−^ mice were viable, we intercrossed them to produce compound and double knockout mice. Mice null for either *Cited2* or *Lmo4* were not present at weaning, as expected (Supp. Table S2). Embryos from *Lmo4*^+/−^;*Cited2*^+/−^ intercrosses were collected at E15.5 and examined by MRI (n = 141). Supplementary Table S3 and Supp. Figures S1 and S2 summarize the defects observed in these embryos, the genotypes of which were all present in normal Mendelian ratios at this point in gestation. We observed no significant differences in the incidence of cardiovascular malformations in the compound heterozygous/knockout or double knockout embryos. Spina bifida was occasionally observed in embryos of the same genotype that exhibited exencephaly (Supp. Fig. 1), a phenotype not described before for either *Lmo4* or *Cited2* mutant embryos.

**Figure 4:**
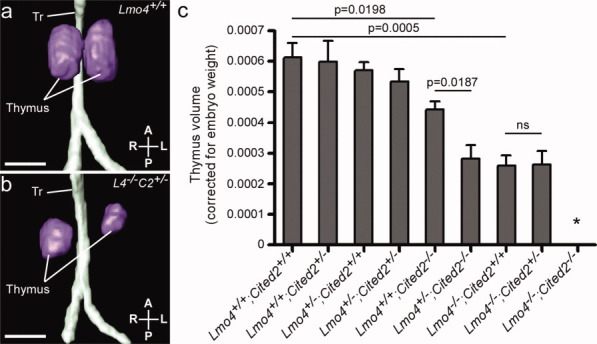
Impaired thymus development in *Lmo4* and *Cited2* compound mutant embryos. a,b: Three-dimensional (3D) reconstruction of thymuses from embryonic day (E) 15.5 embryos by MRI. **a:** Thymus and trachea from a wild-type (*Lmo4*^+/+^) embryo, showing normal position and equal-sized lobes. **b:***Lmo4*^−/−^;*Cited2*^+/−^ thymus illustrating hypoplasia, abnormal positioning, and separation of the lobes. **c:** Comparison of whole thymus volumes, adjusted for embryo weight, in embryos of all possible genotypes from *Lmo4*^+/−^;*Cited2*^+/−^ intercrosses (Mean ± SEM). *Lmo4*^−/−^;*Cited2*^−/−^ embryos do not have a thymus (*). Scale bar = 500 μm.

Although cardiovascular development did not seem to be affected by *Lmo4* and *Cited2* epistasis, the thymus was absent in *Lmo4*^−/−^;*Cited2*^−/−^ embryos. Thymus defects were observed in most *Lmo4;Cited2* compound mutant genotypes (Supp. Table S3), including thymus hypoplasia, separated thymus lobes, and one lobe absent (Fig. 4a,b). A single *Lmo4*^−/−^;*Cited2*^+/−^ embryo was also lacking both thymus lobes. Thymuses from representative embryos of all genotypes were compared by volume (n = 5 each genotype; Fig. 4c). No significant difference was found between the thymuses of *Lmo4*^+/−^;*Cited2*^+/+^, *Lmo4*^+/+^;*Cited2*^+/−^ or *Lmo4*^+/−^;*Cited2*^+/−^ embryos when compared with thymuses from wild-type embryos. The thymuses of *Lmo4*^−/−^;*Cited2*^+/+^ (*P* = 0.0005) and *Lmo4*^+/+^;*Cited2*^−/−^ (*P* = 0.0198) embryos were significantly reduced compared with those of wild-type embryos. Further loss of one copy of *Lmo4* reduced the average thymus volume in *Lmo4*^+/−^;*Cited2*^−/−^ embryos compared with *Lmo4*^+/+^;*Cited2*^−/−^ embryos (*P* = 0.0187), but thymuses of *Lmo4*^−/−^;*Cited2*^+/−^ embryos, when present, were not significantly smaller than in *Lmo4*^−/−^;*Cited2*^+/+^ embryos.

The complete lack of thymus in *Lmo4*^−/−^;*Cited2*^−/−^ embryos is a novel phenotype, not previously seen in either individual knockout. This indicates that *Cited2* and *Lmo4* interact to control thymus development. To investigate whether there is a common target for *Cited2* and *Lmo4*, embryos were harvested for quantitative reverse transcriptase-polymerase chain reaction (QRT-PCR) analysis. Pharyngeal arches and hearts were dissected from E10.5 *Cited2*^−/−^ and *Lmo4*^−/−^ embryos. Expression of *Cited2* and *Lmo4*, as well as potential target candidate genes, *Pitx2c* and *Tbx1*, were investigated. *Pitx2c* was selected as it is a known target of *Cited2* (Bamforth et al., 2004), and *Tbx1* was selected as it has been implicated in thymus development (Jerome and Papaioannou, 2001).

### *Tbx1* Expression in *Cited2*^−/−^ and *Lmo4*^−/−^ Embryos

**Figure 5:**
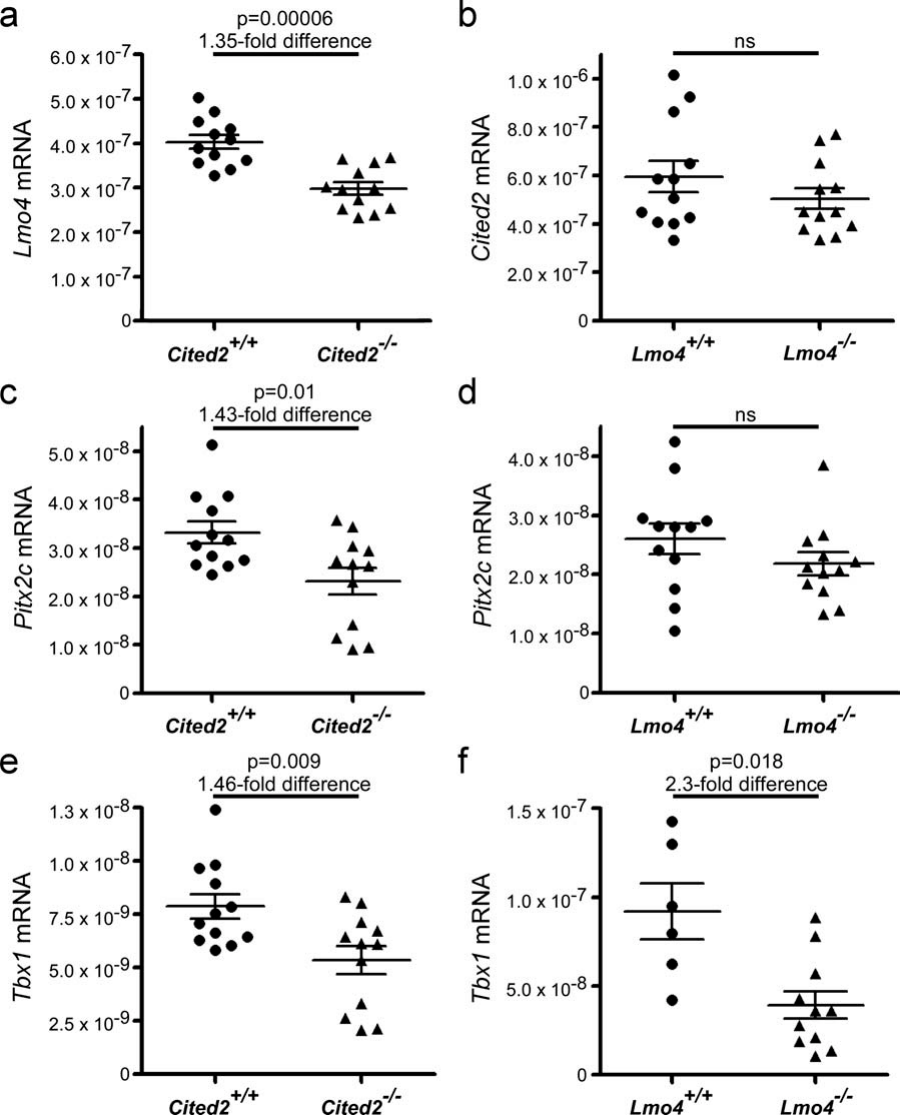
mRNA expression in embryos lacking *Cited2* or *Lmo4*. Quantitative reverse transcriptase-polymerase chain reaction was conducted on pharyngeal arches and hearts harvested from embryonic day (E) 10.5 *Cited2*^+/+^, *Cited2*^−/−^, *Lmo4*^+/+^ and *Lmo4*^−/−^ embryos. **a:***Lmo4* expression is significantly decreased in *Cited2*^−/−^ embryos. **b:***Cited2* expression was not affected in *Lmo4*^−/−^ embryos. **c,d:***Pitx2c* expression was significantly decreased in *Cited2*^−/−^ embryos (c) but was not affected in *Lmo4*^−/−^ embryos (d). **e,f:***Tbx1* expression was reduced in *Cited2*^−/−^ embryos (e) and in *Lmo4*^−/−^ embryos (f). Mean ± SEM are indicated.

Gene expression levels were analyzed from the dissected hearts and pharyngeal arches of E10.5 embryos. *Cited2*^−/−^ embryos showed a modest yet highly significant decrease in levels of *Lmo4* mRNA when compared with wild-type littermates (1.35-fold decrease; *P* = 0.00006, n = 12 *Cited2*^+/+^ vs. n = 12 *Cited2*^−/−^ embryos), but *Lmo4*^−/−^ embryos did not show any difference in *Cited2* levels (n = 12 *Lmo4*^+/+^ vs. n = 12 *Lmo4*^−/−^ embryos; Fig. 5a,b), indicating that *Lmo4* is downstream of *Cited2*. As we have previously shown (MacDonald et al., 2008), *Cited2*^−/−^ embryos have lower levels of *Pitx2c* mRNA, compared with wild-type littermates (1.43-fold decrease, *P* = 0.01; Fig. 5c), but *Lmo4*^−/−^ embryos did not show any difference in *Pitx2c* levels (n = 12 embryos for each genotype tested; Fig. 5d). *Tbx1* levels were then investigated in the dissected pharyngeal arch regions from E10.5 *Cited2*^−/−^ and *Lmo4*^−/−^embryos. A significant decrease in *Tbx1* levels was found in both *Cited2*^−/−^ (1.46-fold decrease, p = 0.009) and *Lmo4*^−/−^ (2.3-fold decrease; *P* = 0.018, n = 12 embryos for each genotype tested; Fig. 5e,f). This suggests that *Tbx1* could be a common target for *Cited2* and *Lmo4* in the developing pharyngeal arches.

## DISCUSSION

Genetic interactions can be identified through screens testing pairwise combinations of mutant genes that either suppress or enhance the original mutant phenotype (Hartman et al., 2001). In pairwise intervention experiments, the expected outcome of a double-intervention is the product of the two single-intervention effects; departure from this model indicates a functional relationship (i.e., either an aggravating or an alleviating effect, indicating compensatory function or gene-action in series, respectively) between the two target genes (St Onge et al., 2007). This approach has been used in *Drosophila melanogaster* and *Caenorhabditis elegans* to identify genes acting in the same pathway (Lehner et al., 2006; Sambandan et al., 2008). Also, genes that share similar or identical GO term annotations are more likely to interact genetically, as shown by the analysis of thousands of mutant *Saccharomyces cerevisiae* yeast strains (Wong et al., 2004). This is also true for genes that encode proteins found in the same subcellular location, or that act in the same protein complex. Another excellent predictor of a genetic interaction is a shared specific mutant phenotype (Tong et al., 2004). Mouse embryos lacking *Cited2* or *Lmo4* share specific phenotypes, both displaying exencephaly and cardiovascular, thymus, skeletal, and cranial ganglia defects. Experiments to identify a genetic interaction revealed that *Lmo4*^−/−^;*Cited2*^−/−^ embryos completely lacked a thymus, a novel phenotype, not observed in either parental line. However, there was no significant increase in penetrance of cardiac malformation in *Lmo4*^−/−^;*Cited2*^−/−^ embryos.

As discussed by Wong et al., a genetic interaction between two genes is indicated when the phenotype observed is more pronounced than the phenotype produced by the genes individually (Wong et al., 2004). Having re-analyzed previously published studies, Mani et al. (2008) concluded that the expected phenotype in offspring should be the product of the parental fitness, and not the average of parental fitness or that of the least fit parent as previously thought (Tong et al., 2001, 2004). This allows for greater expected deviation from parental fitness in offspring. For instance, if one parent had a fitness of 0.6 and one a fitness of 0.8, the expected fitness of the offspring is not 0.7 (average) or 0.6 (least fit parent), but 0.48 (product). Thus, applying this principle to these results, where *Lmo4*^−/−^ embryos had thymuses 0.46 times the size of wild-type thymuses, and *Cited2*^−/−^ thymuses were 0.78 times the size of wild-type thymuses, the expected thymus size of *Lmo4*^−/−^;*Cited2*^−/−^ embryos should be 0.36 times the size of those in wild-type embryos. However, a total lack of thymus was discovered, dramatically exceeding the expected decrease. This indicates that a genetic interaction between *Lmo4* and *Cited2* is necessary for thymus development, suggesting that the genes act together in a common developmental process or molecular target.

*Pitx2c* is a known target of *Cited2*, but QRT-PCR data showed that it is not a target of *Lmo4*. Nor is *Cited2* a target of *Lmo4*, although it appears that *Lmo4* could be a target of *Cited2*. The data also revealed that *Tbx1* expression levels are reduced in response to loss of both *Cited2* and *Lmo4* expression. This could indicate that *Tbx1* is a common target of *Cited2* and *Lmo4*, all of which are necessary for normal thymus formation. Indeed, Ivins et al. identified a reduction in *Lmo4* expression in embryos lacking *Tbx1* expression at E9.5 (Ivins et al., 2005). *Tbx1* is expressed in the developing pharyngeal endoderm, which contributes to thymus formation. Absent thymus is a feature of DiGeorge / 22q11 deletion syndrome (Lischner, 1972; de la Chapelle et al., 1981), for which the majority of phenotypes observed are largely attributed to haploinsufficiency of *TBX1* during development (Chieffo et al., 1997; Jerome and Papaioannou, 2001; Lindsay and Baldini, 2001). Moreover, in the mouse, morphology of the thymus is sensitive to *Tbx1* dosage (Zhang and Baldini, 2007). *Lmo4* is strongly expressed in the pharyngeal arches during mouse embryonic development (Hahm et al., 2004), specifically in the migratory cranial neural crest, and highly expressed in proliferating T lymphocytes in adult thymus (Kenny et al., 1998). *Cited2* is also strongly expressed in the pharyngeal arches during development, in endoderm, mesenchyme and ectoderm (Weninger et al., 2005; MacDonald et al., 2008), and it is the third pharyngeal pouch from which the thymus is derived. The expression of *Tbx1* in the fourth pharyngeal arch is also important for normal formation of the fourth pharyngeal arch arteries (Lischner, 1972; Jerome and Papaioannou, 2001), the development of which are affected in embryos lacking *Cited2* and/or *Lmo4*.

Our data show that *Lmo4*^−/−^ embryos have incomplete penetrance of cardiovascular defects in addition to those phenotypes already known, which has not been previously reported. Thymus defects have also not been previously reported and were detected by MRI, despite Tse et al. (2004) noting a normal cellular architecture of the thymus from histological sections, and no hypoplasia was recorded (Tse et al., 2004). Adding cardiovascular and thymus defects to the list of similarities between the recorded phenotypes of both *Cited2* and *Lmo4* knockout mice gave strong grounds for a physical and genetic interaction between *Cited2* and *Lmo4*. Indeed, using GST pull-down assays, we were able to show that LMO4 physically interacts with residues 123–161 of CITED2, although we were unable to co-immunoprecipitate Lmo4 and Cited2 (data not shown).

To fully understand the genetic network in which *Cited2*, *Lmo4*, and *Tbx1* are potentially interacting to control thymus development, further experiments will need to be performed. Although QRT-PCR data gives a quantitative measure of altered expression, qualitative assays will be needed to complement this, for example, by using in situ hybridization techniques to investigate whether the expression of *Tbx1* is reduced specifically in the endoderm as may be expected. A more detailed investigation of candidate genes in wild-type and double heterozygote embryos and double homozygous null embryos will also need to be carried out, looking at the expression profile of genes specifically expressed in thymus such as *Foxn1* (Nehls et al., 1996), and those expressed in the pharyngeal arches and known to affect thymus development, for example, *Pax9*, *Eya1*, and *Six1* (Peters et al., 1998; Xu et al., 2002; Zou et al., 2006).

To summarize, in this study we have shown that mice lacking *Lmo4* exhibit cardiovascular and thymus defects, which are novel phenotypes and add *Lmo4* to the genetic network involved in cardiovascular development. *Lmo4* interacts genetically with *Cited2* in vivo to control thymus development, possibly through a common target gene in *Tbx1*.

## EXPERIMENTAL PROCEDURES

### Mice

*Cited2*^+/−^ mice (*Cited2*^*tm1Bha*^) on a mixed or congenic C57BL/6J background (Bamforth et al., 2001, 2004) and *Lmo4*^+/−^ mice (gift from T. Rabbitts, LMB Cambridge; Tse et al., 2004) were used in this study. Embryos were harvested at the indicated time points after detection of a vaginal plug (E0.5), and genotyped using allele-specific PCR (primer details are available on request). All animal experimentation was performed under UK Home Office authorization and regulations.

### Imaging

Magnetic resonance imaging was performed on a horizontal 9.4T/21cm VNMRS Direct Drive MR system (Varian Inc., Palo Alto) essentially as described previously (Schneider et al., 2004) on embryos at E15.5. Skeletons were prepared and stained with alizarin red (bone) and Alcian blue (cartilage) by standard methods.

### QRT-PCR

The hearts and pharyngeal arch regions were dissected free from E10.5 embryos as described (Prescott et al., 2005). RNA isolation, cDNA synthesis, and quantitative RT-PCR (QRT-PCR) reactions were carried out as described (MacDonald et al., 2008) using preoptimized TaqMan primer-probe sets from Applied Biosytems (*Mus musculus* assays Mm00516121_m1 (NM_010828 Cited2), Mm00495373_m1 (NM_010723 Lmo4), Mm00440826_m1 (NM_001042502 Pitx2c), Mm00448948_m1 (NM_011532 Tbx1), and eukaryotic 18S rRNA. Expression levels were normalized to 18S rRNA using the R_0_ method of analysis (Peirson et al., 2003).

### Statistical Analyses

The chi-squared test was used to calculate the deviation from expected Mendelian ratios. QRT-PCR data was analyzed using a two-tailed, two-sample *t*-test assuming unequal variance.

## Supplementary material

Supp. Fig. S1. Neural tube, cleft palate, and cardiac defects observed in mutant embryos. Embryos at embryonic day (E) 15.5 generated by intercrossing Lmo4+/−;Cited2+/− mice were examined. a: Normal wild-type embryo. b: Lmo4−/−;Cited2+/− embryo with exencephaly (Ex) and spina bifida (SB). Scale bar = 1 mm. c–l: Two-dimensional images generated by magnetic resonance imaging. c,d: Sagittal sections through wild-type and Lmo4−/−;Cited2+/− embryos. c: Wild-type embryo with complete palate (P) over the tongue (T). d: Lmo4−/−;Cited2+/− embryo with cleft palate (cP) and edema (E). e,f: Coronal sections of a normal wild-type (e) and abnormal Lmo4−/−;Cited2−/− (f) heart. g,h: Coronal sections of organs from normal wild-type (g) and abnormal Lmo4+/−;Cited2−/− (h) embryo. The mutant displays right pulmonary isomerism and right-sided stomach. i–l: Transverse sections of hearts. i: Normal wild-type heart. j: Lmo4+/−;Cited2−/− heart with a common atrium (A) and a primum atrial septal defect (ASDP). A common atrioventricular valve (AVV) opens into both ventricles. k: Lmo4+/−;Cited2−/− heart with a common atrium (A) and heart apex pointing to the right. l: Lmo4−/−;Cited2+/+ heart positioned severely to the left. IVS, interventricular septum; LV, left ventricle; MV, mitral valve; PAS, primary atrial septum; RV, right ventricle; TV, tricuspid valve; WT, wild-type. Scale bar = 500 μm.

Supp. Fig. S2. Cardiovascular defects observed in mutant embryos. Embryos at embryonic day (E) 15.5, generated by intercrossing Lmo4+/−;Cited2+/− mice, were examined by magnetic resonance imaging (MRI), and three-dimensional (3D) reconstructions were made. a–f: Ventral views. a: Wild-type (WT) heart showing normal topology. b: Lmo4−/−;Cited2+/− heart showing a ventricular septal defect (VSD) and double outlet right ventricle (DORV). The ductus arteriosus is right-sided. c: Lmo4−/−;Cited2+/− heart showing right-sided aortic arch (RAA) and ductus arteriosus (RDA), and a mirror image arrangement of the pharyngeal arch arteries. The heart is twisted severely to the left. d: Lmo4+/−;Cited2−/− heart showing an interrupted aortic arch (IAA) and DORV, with an aberrant right subclavian artery (ARSA). e: Lmo4−/−;Cited2−/− heart with a common arterial trunk (CAT). f: Lmo4+/−;Cited2−/− heart with CAT, RAA, and VSD. g,h: Dorsal views. g: Wild-type (WT) heart showing normal topology of venous return to the heart. h: Lmo4+/−;Cited2−/− heart showing a common atrium (A) with bilateral systemic venous sinuses (LSVS, RSVS) into which drain bilateral superior and inferior caval veins. Scale bar = 500 μm.
