# Supplementary figures and images for: A Novel Role for Transcription Factor Lmo4 in Thymus Development Through Genetic Interaction with Cited2
Source: Dev Dyn. 2010 May 28;239(7):1988–94. doi: 10.1002/dvdy.22334 (PMC3417300; doi:10.1002/dvdy.22334)

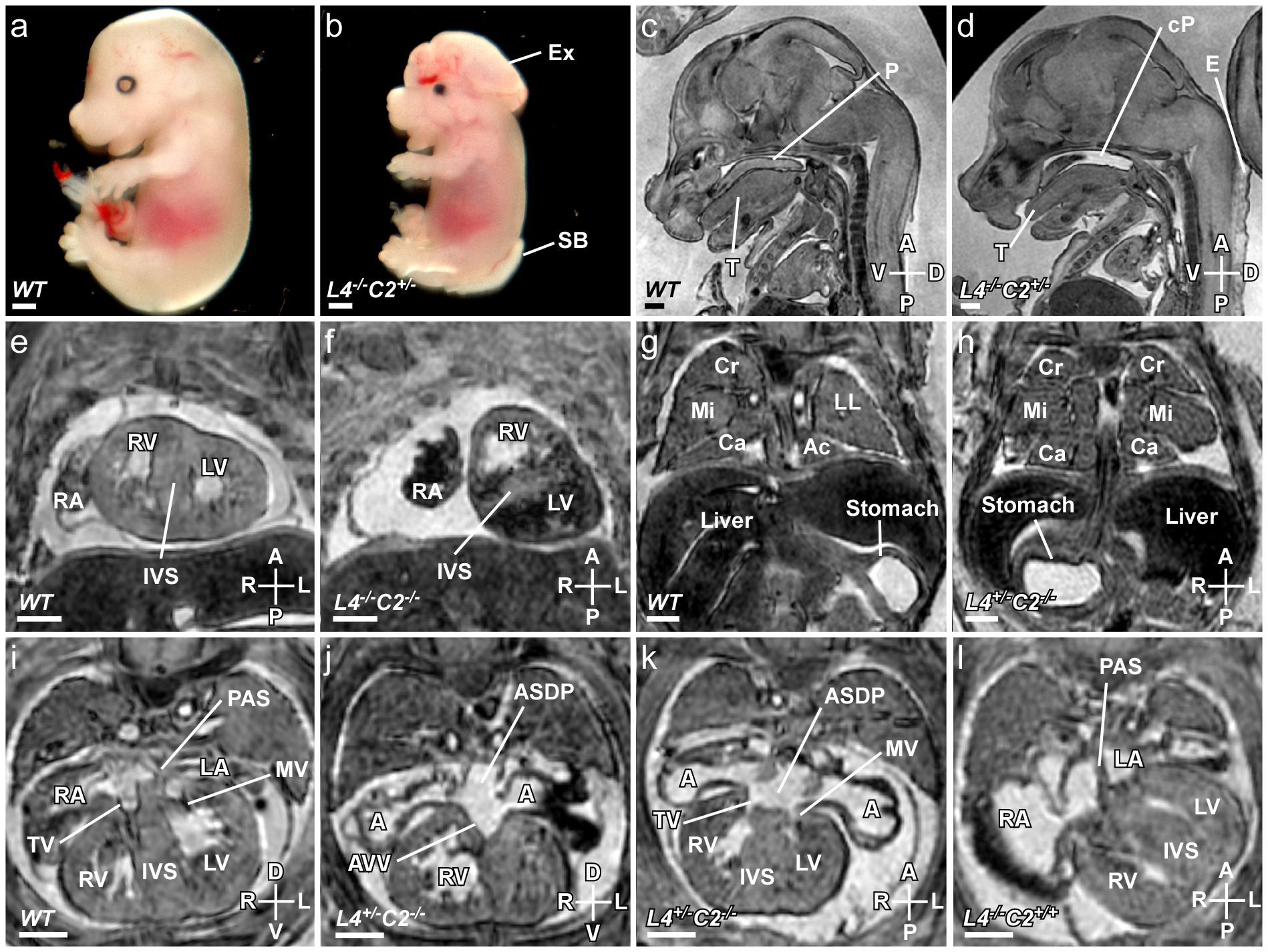

Supplement: Supplementary file 1 [file dvdy0239-1988-SD1.tif]

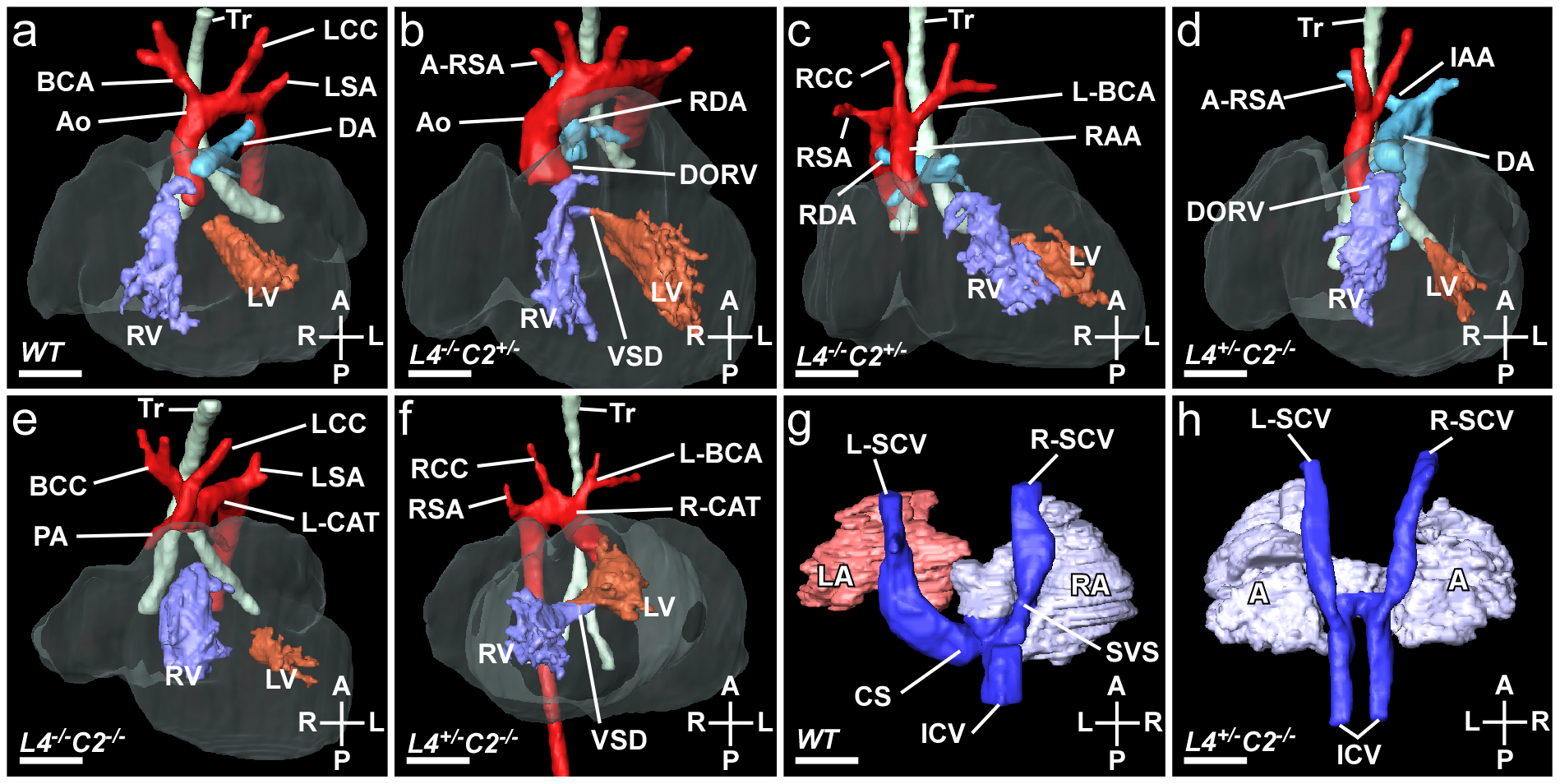

Supplement: Supplementary file 2 [file dvdy0239-1988-SD2.tif]
