# Supplementary material for: A Novel Role for Transcription Factor Lmo4 in Thymus Development Through Genetic Interaction with Cited2
Source: Dev Dyn. 2010 May 28;239(7):1988–94. doi: 10.1002/dvdy.22334 (PMC3417300; doi:10.1002/dvdy.22334)
Supplement: Supplementary file 3 [file dvdy0239-1988-SD3.doc]

**SUPPLEMENTARY DATA**

<SUPTBLS1>Supp. Table 1. Genotypes of mice at weaning produced from the *Lmo4^+/—^* and *Cited2^+/—^* intercross.

| Genotype | n | Expected n |
| --- | --- | --- |
| *Lmo4^+/+^*;*Cited2^+/+^* | 57 | 49.25 |
| *Lmo4^+/—^*;*Cited2^+/+^* | 62 | 49.25 |
| *Lmo4^+/+^*;*Cited2^+/—^* | 38 | 49.25 |
| *Lmo4^+/—^*;*Cited2^+/—^* | 40 | 49.25 |

Four possible genotypes are expected from this cross. The number of pups observed for each genotype (n) and the expected Mendelian number relative to the total number of mice examined (n = 197) is presented. A chi-squared test shows that this distribution differs significantly from expected (χ^2^ value 8.8, d.f. 3, p = 0.032).</SUPTBLS1>

<SUPTBLS2>Suppl. Table 2. Genotypes of Mice at Weaning Produced From the *Lmo4^+/—^*;*Cited2^+/—^* Intercross

| Genotype | | n | Expected no. |
| --- | --- | --- | --- |
| *1* | *Lmo4^+/+^*;*Cited2^+/+^* | 8 | 6.6 |
| *2* | *Lmo4^+/—^*;*Cited2^+/+^* | 25 | 13.3 |
| *3* | *Lmo4^+/+^*;*Cited2^+/—^* | 7 | 13.3 |
| *4* | *Lmo4^+/—^*;*Cited2^+/—^* | 20 | 26.6 |
| *5* | *Lmo4^—/—^*;*Cited2^+/+^* | 0 | 0 |
| *6* | *Lmo4^+/+^*;*Cited2^—/—^* | 0 | 0 |
| *7* | *Lmo4^—/—^*;*Cited2^+/—^* | 0 | 0 |
| *8* | *Lmo4^+/—^*;*Cited2^—/—^* | 0 | 0 |
| *9* | *Lmo4^—/—^*;*Cited2^—/—^* | 0 | 0 |

Nine possible genotypes are expected from this cross, although genotypes 5&ndash;9 would not be expected to survive. The number of pups observed for each genotype (n) and the expected Mendelian number relative to the total number of mice examined (n = 60) is presented. A chi-squared test shows that this distribution differs significantly from expected (χ^2^ value 15.15, d.f. 3, <I>P</I> = 0.002).</SUPTBLS1>

<SUPTBLS1>Supp. Table 3. Summary of Defects Observed in E15.5 Embryos Generated by Intercrossing *Lmo4^+/—^;Cited2^+/—^* Mice

| Genotype | | Embryo  *n* | Heart | | | | Thymus | | | Cleft palate | Spina bifida | Exen-cephaly |
| --- | --- | --- | --- | --- | --- | --- | --- | --- | --- | --- | --- | --- |
| *Lmo4* | *Cited2* |  | AoA | Sept | OFT | LR | Sm | ½ | Ab |  |  |  |
| wt | wt | 10 | 0 | 0 | 0 | 0 | 0 | 0 | 0 | 0 | 0 | 0 |
| wt | het | 22 | 1 | 1 | 1 | 1 | 2 | 0 | 0 | 1 | 0 | 0 |
| wt | null | 7 | 1 | 5 | 6 | 2 | 2 | 1 | 0 | 1 | 1 | 2 |
| het | wt | 24 | 0 | 1 | 1 | 0 | 2 | 0 | 0 | 1 | 0 | 0 |
| het | het | 29 | 1 | 0 | 0 | 0 | 0 | 0 | 0 | 1 | 0 | 0 |
| het | null | 13 | 9 | 9 | 13 | 7 | 6 | 5 | 0 | 4 | 1 | 6 |
| null | wt | 8 | 7 | 7 | 6 | 0 | 6 | 1 | 0 | 7 | 0 | 0 |
| null | het | 23 | 16 | 20 | 21 | 0 | 13 | 4 | 1 | 10 | 2 | 8 |
| null | null | 5 | 5 | 4 | 5 | 3 | 0 | 0 | 5 | 0 | 1 | 4 |

Genotype and total number of embryos with each genotype are specified on the left-hand side; table shows the number of embryos of each genotype afflicted by a particular phenotype. Heart = any heart defects, divided into aortic arch (AoA; including interrupted, right-sided or hypoplastic aortic arch, abnormal carotid artery, abnormal subclavian artery, vascular ring), septal (Sept; including atrial and ventricular septal defects), outflow tract (OFT; including double outlet right ventricle, common arterial trunk, abnormal pulmonary trunk morphology, abnormal pulmonary artery morphology, overriding aorta), and left&ndash;right patterning (LR; including right atrial isomerism, dextrocardia, abnormal ventricular topology. Left and right pulmonary isomerisms and situs ambiguous are also included in this definition). Thymus = any thymus defect including small or separated/malpositioned lobes (Sm), one lobe missing (half), or totally absent (Ab). The incidence of cleft palate, spina bifida, and exencephaly are also recorded. A chi-squared test shows that the distribution of embryo genotypes does not differ significantly from expected (χ^2^ value 9.61, d.f. 8, p = 0.2935).</SUPTBLS1>
